# Novel and traditional lipid profiles in Metabolic Syndrome reveal a high atherogenicity

**DOI:** 10.1038/s41598-019-48120-5

**Published:** 2019-08-13

**Authors:** Sílvia Paredes, Liliana Fonseca, Laura Ribeiro, Helena Ramos, José Carlos Oliveira, Isabel Palma

**Affiliations:** 1 Sílvia Cristina de Sousa Paredes. Endocrinology Department, Hospital de Braga, Sete Fontes, São Victor, 4710-243 Braga Portugal; 2 Liliana Cecília Martins da Fonseca. Endocrinology Department, Centro Hospitalar e Universitário do Porto, Largo Professor Abel Salazar, 4099-001 Porto, Portugal; 3 Laura Virgínia Pereira Teixeira Ribeiro. Department of Public Health and Forensic Sciences, and Medical Education, Medical Education Unit, Faculty of Medicine of the University of Porto, 4200-319 Porto, Portugal; 4 I3S-Instituto de Investigação e Inovação em Saúde, Universidade do Porto, 4200-319 Porto, Portugal; 5 Maria Helena da Silva Ramos. Endocrinology Department, Centro Hospitalar e Universitário do Porto, Largo Professor Abel Salazar, 4099-001 Porto, Portugal; 6 José Carlos Azevedo Oliveira. Clinical Chemistry Department, Centro Hospitalar e Universitário do Porto, Largo Professor Abel Salazar, 4099-001 Porto, Portugal; 7 Isabel Maria Gonçalves Mangas Neto da Palma. Endocrinology Department, Centro Hospitalar e Universitário do Porto, Largo Professor Abel Salazar, 4099-001 Porto, Portugal

**Keywords:** Dyslipidaemias, Metabolic syndrome, Dyslipidaemias

## Abstract

Low-density-lipoprotein cholesterol (LDL-c) guides lipid-lowering therapy, although other lipid parameters could better reflect cardiovascular disease (CVD) risk. Discordance between these parameters and LDL-c has not been evaluated in metabolic syndrome (MetS) patients. We characterized a comprehensive lipid profile in 177 MetS patients. The 2016 ESC/EAS Guidelines for the Management of Dyslipidemias were used to define LDL-c targets. The atherogenic lipoprotein profile was compared in patients with LDL-c within and above the target. Only 34.4% (61) of patients had mean LDL-c levels within the guidelines and patients with LDL-c above target presented significantly elevated levels of Apolipoprotein B (ApoB), non-high-density lipoprotein cholesterol (non-HDL-c) and oxidized LDL-c. In patients with LDL-c within target, 25%, 31% and 49% presented levels above the recommended range for ApoB, non-HDL-c and oxidized LDL-c, respectively. Patients presented a strong association of LDL-c and non-HDL-c (r = 0.796), ApoB (r = 0.749) and oxidized LDL-c (r = 0.452). Similarly, non-HDL-c was strongly correlated with ApoB (r = 0.857) and oxidized-LDL-c (r = 0.555). The logistic regression model evidenced higher triglycerides and HDL-c and lower ApoB as predictors of having LDL-c within target. Reliance solely on LDL-c could result in missed opportunities for CVD risk reduction. ApoB, oxidized LDL-c, and particularly non-HDL-c, could be valuable parameters to estimate the CVD risk of MetS patients and have the potential to be targeted therapeutically.

## Introduction

The importance of low-density lipoprotein (LDL) cholesterol (LDL-c) reduction to prevent cardiovascular (CV) disease (CVD) is strongly acknowledged^1,2^. Although LDL-c is recommended as the primary marker to guide lipid-lowering therapy^3^, some studies have suggested that other lipid parameters may better represent coronary heart disease (CHD) risk^4,5^. In fact, the 2016 European Society of Cardiology /European Atherosclerosis Society (ESC/EAS) Guidelines for the management of dyslipidemias advocates that non-high-density lipoprotein cholesterol (non-HDL-c) and Apolipoprotein B (ApoB) should be evaluated and considered secondary targets for lipid control^3^. This is in accordance with a growing body of evidence suggesting that other lipid parameters such as non-HDL-c or ApoB may provide a more accurate measure of CHD risk in comparison to LDL-c^4,5^.

Patients with metabolic syndrome (MetS), a group at high risk of developing CVD, show several lipid abnormalities other than elevated LDL-c, namely elevated triglycerides levels, low high-density lipoprotein (HDL) cholesterol (HDL-c) and high levels of small and dense LDL (sd-LDL) particles^6^. Thus, evidence suggests that measuring LDL-c may be insufficient in these patients.

The aim of this study was to evaluate a comprehensive lipid profile in MetS patients and compare those achieving and not achieving LDL-c control in respect to other non-conventional lipid parameters.

## Materials and Methods

### Subjects and study design

We performed a retrospective data evaluation of our institution’s database and electronic medical records system for patients included in the outpatient department at a Portuguese University Hospital, with information regarding an extended lipid profile. Patients followed in the Dyslipidemia and Dermatology outpatient departments between January 2010 and September 2017 were included. After analysis of the medical records of 345 patients, 177 patients with the diagnosis of MetS were included in this study.

### Data collection

Obesity was defined as a body mass index (BMI) ≥30 kg/m^2^ and overweight as BMI ≥25 and <30 kg/m^2^^7^. Hypertension was defined as systolic blood pressure ≥140 mmHg and/or diastolic blood pressure ≥90 mmHg on at least two blood pressure measurements per visit and on at least two visits^8^ and/or prescription of any antihypertensive medication. Diabetes was defined as plasma glucose ≥126 mg/dL in at least two measurements, A1c ≥6.5% or prescription of any antidiabetic medication^9^. Prediabetes was defined as a plasma glucose ≥100 and <126 mg/dL^9^. MetS was classified using the 2009 update: abdominal obesity (waist circumference ≥94 cm for men and ≥80 cm for women)^10^; triglycerides ≥150 mg/dL or the presence of drug treatment for elevated triglycerides; HDL-c <40 mg/dL in men and <50 mg/dL in women or the presence of a drug treatment for reduced HDL-c; systolic blood pressure ≥130 mmHg and/or diastolic blood pressure ≥85 mmHg or the presence of an antihypertensive drug; fasting glucose ≥100 mg/dL or the presence of a lowering-glucose drug^10^. Any 3 of 5 risk factors constitutes a diagnosis of MetS^10^.

Patients were classified as smokers if they consumed any quantity of tobacco. A former smoker was defined as having quit smoking at least 6 months before assessment. Alcohol consumption was considered when patients reported drinking daily at least 1 or 2 glasses of an alcoholic beverage, for women and men respectively.

Patients with conditions that could interfere with the lipid profile, such as genetic dyslipidemia, hepatic or renal moderate to severe disease, cancer, viral infection (hepatitis B or C, HIV), genetic metabolic disease, hyper or hypothyroidism were excluded. Pediatric patients and pregnant women were also excluded as well as patients treated with drugs that could exacerbate lipid profile, such as glucocorticoids.

In order to stratify risk categories, patients were classified as having “very high risk”, “high risk”, “moderate risk” or “low risk” accordingly to the 2016 ESC/EAS Guidelines for the Management of Dyslipidemias^3^. The 10-year risk of fatal CVD was calculated using Systemic Coronary Risk Estimation (SCORE). LDL-c targets were defined according to the following ESC/EAS Guidelines^3^, LDL-c <70 mg/dL for very high-risk patients, LDL-c <100 mg/dL for high risk and LDL-c <115 mg/dL for low to moderate risk. In order to analyze other lipid parameters and their agreement with LDL-c levels, targets were also defined for these parameters. The 2016 ESC/EAS Guidelines for the Management of Dyslipidemias was used to define targets such as ApoB, Apolipoprotein A1 (ApoA1), non-HDL-c and Lipoprotein (a) [Lp(a)]^3^: ApoB: <80 mg/dL in very high risk individuals and <100 mg/dL in high risk individuals; Non-HDL-c: <100 mg/dL in very high risk individuals, <130 mg/dL in high risk individuals and <145 mg/dL in low to moderate risk individuals; ApoA1: low if <120 mg/dL in men and <140 mg/dL in women; Lp(a) <50 mg/dL. For the remaining parameters, the following laboratory reference values were used: oxidized LDL-c: 26–117 mg/dL; ApoB/ApoA1 ratio: 0.45–1.25 and sd-LDL particles: <35 mg/dL.

### Laboratory analysis

Biochemical laboratory tests were conducted after an 8-hour night fast. Lipid parameters included total cholesterol, HDL-c, triglycerides, ApoB, ApoA1, oxidized LDL-c, Lp(a) and sd-LDL. LDL-c levels were calculated through Friedewald’s formula^11^: LDL-c (mg/dL) = total cholesterol (mg/dL) − HDL-c (mg/dL) – triglycerides (mg/dL)/5, unless triglycerides ≥400 mg/dL, where direct LDL-c measurement was performed. Non-HDL-c was calculated by subtracting HDL-c to total cholesterol. Other parameters such as creatinine, uric acid, glucose and homocysteine were also evaluated. A1c was assessed in the majority of diabetic patients. Creatinine, glucose, uric acid, total cholesterol, HDL-c and triglycerides were measured using an enzymatic colorimetric method, with intra and inter-assay coefficients of variation of <2.8% and <3.9%, respectively. These parameters were measured using an automated autoanalyzer (Cobas 8000, Roche Diagnostics, Mannheim, Germany). ApoA1, ApoB and Lp(a) were evaluated through immunoturbidimetry, with intra and inter-assay coefficients of variation of <1% and <2.4% for ApoA1, <1.2% and <3.2% for ApoB and <3.7% and <3.8% for Lp(a), respectively. These parameters were evaluated using an automated autoanalyzer (Cobas Integra 400, Roche Diagnostics, Mannheim, Germany). Oxidized LDL-c was measured by Sandwich Enzyme-Linked ImmunoSorbent Assay (Mercodia, Uppsala, Sweden), with intra and inter-assay coefficients of variation of <7.3% and <8.3%. Sd-LDL particles were measured using an enzymatic colorimetric method (Randox Laboratories, UK) with intra and inter-assay coefficients of variation of <3% and <3%. A1c was evaluated using an affinity chromatography method (Variant II turbo, BioRad Laboratories, CA, USA), with intra and inter-assay coefficients of variation of <0.78% and <0.66%. Homocysteine was measured by nephelometry (Dimension Vista 500, Siemens Healthcare, Germany), with intra and inter-assay coefficients of variation of <3.3% and <8.2%.

### Statistical analysis

Statistical analysis was performed using IBM SPSS® version 25.0 and a p value below 0.05 was considered statistically significant. For continuous quantitative variables, distribution normality was tested through histogram observation and kurtosis and skewness analysis. Results are presented as mean values ± standard-deviation and median values (25–75 percentiles). The chi-square test was used to analyze differences between groups in categorical variables. The Student t-test for independent variables and the Mann Whitney test were used to compare continuous variables with normal and non-normal distribution between groups, respectively. Correlations were evaluated using the Pearson and the Spearman correlation tests for continuous symmetrical and asymmetrical variables, respectively, and applying a Bonferroni correction. A logistic regression model was performed in order to evaluate predictors of LDL-c within target, adjusting for potential confounders using a stepwise regression with a forward inclusion approach.

### Ethics

This study was approved by the local Ethics committee (150-DEFI/149-CES) and procedures were carried out in accordance with the principles contained in the Declaration of Helsinki. Due to the retrospective nature of this study, consent to participate was waived by the Ethics Committee.

## Results

This study enrolled 177 subjects (99 men and 78 women) with a mean age of 54 ± 12 years. The clinical characteristics of the patients are presented in Table 1. Mean LDL-c levels were 106.4 ± 43.4 mg/dL. Diabetes was present in 97 individuals (54.8%) and the majority of patients (n = 94, 53.1%) were under lipid-lowering drugs and statins were the most common. Twenty-eight (15.8%) individuals were on fibrates and 11 (6.2%) on ezetimibe. Patients were not taking other drugs for treating dyslipidemia nor any anti-diabetic drugs that could affect the lipid profile such as thiazolidinediones, glucagon-like peptide-1 agonists, dipeptidyl peptidase 4 inhibitors or sodium-glucose co-transporter-2 inhibitors. According to the ESC/EAS guidelines, patients were divided in two groups: patients with LDL-c levels within target (n = 61) and patients with LDL-c levels above target (n = 116). There were no statistically significant differences between the groups regarding gender, age and BMI or between the different features of MetS, namely blood pressure, waist circumference and triglycerides, HDL-c or glucose levels. We found no statistically significant differences between groups in relation to alcohol ingestion, smoking or the use of drugs for dyslipidemia such as statins, fibrates or ezetimibe. Uric acid levels, usually elevated in patients with MetS, and homocysteine levels, a significant predictor of new CV events, were not statistically different between the groups. There was a significant difference between groups with respect to the presence of diabetes.

**Table 1:** Comparison of clinical and laboratory variables between MetS patients with LDL-c within and above target.

	n	LDL-c within target (n = 61)	n	LDL-c above target (n = 116)	*p*
Female n (%)	61	25 (41%)	116	53 (45.7%)	0.549
Age (years)	61	54.6 ± 13.1	116	54.1 ± 11.7	0.790
Body mass index (Kg/m^2^)	47	29.9 ± 3.8	93	30.5 ± 4.8	0.465
Systolic BP (mmHg)	56	140.0 ± 17.0	102	138.2 ± 18.5	0.535
Diastolic BP (mmHg)	45	79.4 ± 12.6	74	80.8 ± 13.6	0.571
Waist circumference (M) (cm)	19	103.5 ± 9.0	23	105.0 ± 11.2	0.653
Waist circumference (F) (cm)	10	104.4 ± 13.6	23	100.3 ± 9.0	0.310
Triglycerides* (mg/dL)	61	174.0 (122.5–302.5)	116	165.5 (110.0–239.2)	0.299
HDL-c (M) (mg/dL)	36	35.8 ± 10.3	63	39.2 ± 10.3	0.117
HDL-c (F) (mg/dL)	25	44.9 ± 11.4	53	43.2 ± 12.1	0.545
Glucose* (mg/dL)	55	102.0 (90.0–125.0)	103	111.0 (94.0–156.0)	0.062
A1c (%)	33	6.5 ± 1.6	65	7.4 ± 1.8	0.024
Uric acid (mg/dL)	41	5.5 ± 1.7	73	5.4 ± 1.7	0.820
Homocysteine (µmol/L)	47	15.8 ± 10.3	92	13.5 ± 6.5	0.171
Diabetes n (%)	60	27 (45%)	115	70 (60.9%)	0.045
Statin use n (%)	56	37 (66.1%)	100	57 (57%)	0.267
Fibrates use n (%)	56	14 (25%)	100	14 (14%)	0.086
Ezetimibe use n (%)	61	4 (6.5%)	115	7 (6.1%)	0.596
Alcohol drinking n (%)	36	11 (30.5%)	63	18 (28.6%)	0.817
Smoking n (%)	54	10 (18.5%)	102	16 (15.7%)	0.897

Data are presented as mean ± standard deviation, unless otherwise indicated by ∗corresponding to data presented as median, 25^th^ and 75^th^ percentiles. LDL-c = Low-density lipoprotein cholesterol; BP = Blood pressure; HDL-c = High-density lipoprotein cholesterol, M = male; F = female

The lipid profile of MetS patients with LDL-c within and above target is presented in Table 2. Mean LDL-c concentration was 71.4 ± 22.6 mg/dL in patients with LDL-c within target and 124.9 ± 40.3 mg/dL in those with LDL-c above target (p < 0.001). The latter group also presented statistically significant elevated levels of total cholesterol, ApoB, non-HDL-c, ApoB/ApoA1 ratio and oxidized LDL-c. No statistically significant differences were found between the two groups regarding ApoA1 and sd-LDL particles.

**Table 2:** Comparison of lipid profile between MetS patients with LDL-c within and above target.

	n	LDL-c within target	n	LDL-c above target	*P*
LDL-c (mg/dL)	61	71.4 ± 22.6	116	124.9 ± 40.3	<0.001^∑^
Total cholesterol (mg/dL)	61	160.6 ± 41.3	116	206.0 ± 59.9	<0.001^∑^
ApoB (mg/dL)	56	80.1 ± 19.9	107	102.9 ± 33.6	<0.001^∑^
ApoA1 (F) (mg/dL)	21	145.5 ± 34.3	50	144.9 ± 31.5	0.939
ApoA1 (M) (mg/dL)	32	133.8 ± 35.0	52	128.3 ± 26.0	0.413
ApoB/ApoA1 ratio	51	0.6 ± 0.2	101	0.8 ± 0.3	<0.001^∑^
Non-HDL-c* (mg/dL)	61	115.0 (95.5–135.0)	116	153.5 (123.0–195.7)	<0.001^∑^
Oxidized LDL-c* (U/L)	61	115.7 (84.8–162.4)	116	153.0 (112.15–216.9)	<0.001^∑^
sd-LDL (mg/dL)	11	28.2 ± 13.2	14	40.7 ± 19.4	0.079
Lipoprotein (a)* (mg/dL)	54	10.0 (4.0–34.5)	103	24.0 (7.0–55.0)	0.027

Data are presented as mean ± standard deviation, unless otherwise indicated by ∗ corresponding to data presented as median, 25^th^ and 75^th^ percentiles. The symbol ∑ represent data that maintain statistical signif*i*cance after Bonferroni correction. LDL-c = Low-density lipoprotein cholesterol; ApoB = Apolipoprotein B; ApoA1 = Apolipoprotein A1; M = male; F = female; Non-HDL-c = non-nigh-density lipoprotein cholesterol; sd-LDL = small and dense LDL-c

Despite having a LDL-c within the goal defined by the guidelines, these patients presented elevated levels of other atherogenic lipoproteins. In fact, 25%, 31.1% and 49.2% of patients presented ApoB, non-HDL-c and oxidized LDL-c levels, respectively above the threshold for CV risk. LDL-c exhibited positive correlations with several lipid particles. After Bonferroni correction, we found statistically significant positive correlations between LDL-c and total cholesterol (r = 0.813, p < 0.001), non-HDL-c (r = 0.796, p < 0.001), ApoB (r = 0.749, p < 0.001), sd-LDL (r = 0.629, p < 0.001), ApoB/ApoA1 ratio (r = 0.462, p < 0.001) and oxidized-LDL-c (r = 0.452, p < 0.001). Non-HDL-c was also significantly and positively correlated with ApoB (r = 0.857, p < 0.001) and oxidized-LDL-c (r = 0.555, p < 0.001). The magnitude of this correlation was stronger for non-HDL-c than for LDL-c.

A logistic regression was performed for the identification of variables that could influence LDL-c levels within, or above, target (Table 3). The higher the tryglicerides and the HDL-c levels, the greater the odds of having LDL-c within target, whereas the odds decreased as the ApoB levels increased.

**Table 3:** Predictors of LDL-c within target.

	OR	95% CI	*p*
Triglycerides	1.011	1.003–1.020	0.010
HDL-c	1.178	1.011–1.373	0.035
ApoB	0.883	0.813–0.959	0.003

Included covariables: age, gender, waist circumference, systolic blood pressure, dyastolic blood pressure, presence of diabetes, presence of obesity, statin use, fibrate use, ApoA1, glucose; HDL-c = High density lipoprotein cholesterol; ApoB = Apolipoprotein B; OR = Odds Ratio; 95% CI = 95% Confidence interval

## Discussion

As CVD is the most common cause of death in the developed world^12^, early identification of individuals at increased CV risk is a priority. Global guidelines recommend LDL-c as the cornerstone of lipid management despite other lipid parameters gaining momentum as indexes for CV risk. Also, the clinical expression of the MetS dyslipidemia has a different pattern since hypercholesterolemia is not the main feature. The present study shows that in MetS several atherosclerotic lipoproteins remain elevated even when the patients have optimal LDL-c levels. Thus, in these patients, the LDL-c measurement may be insufficient to estimate CV risk.

### Non-HDL-c and ApoB

We found that 31% and 25% of MetS individuals had high non-HDL-c and ApoB levels, respectively, regardless of having an LDL-c below the cut off for which treatment initiation is recommended. Since both non-HDL-c and ApoB are considered “secondary targets”, these parameters may not be routinely evaluated, after LDL-c control. Several studies have demonstrated a stronger association for non-HDL-c and ApoB vs LDL-c with CHD events^13,14^, also in MetS patients^15^. Moreover, ApoB and non-HDL-c have been closely associated with MetS development^15,16^ and their levels are higher in MetS patients compared to those without MetS, regardless of the LDL-c level^5,17^. In line with this, the majority of our patients presented elevated ApoB and non-HDL-c levels. It has been demonstrated that in MetS patients or in those with evidence of insulin resistance, a discordance between ApoB and LDL-c is more evident and ApoB appears to be superior to LDL-c in predicting CV risk^18^. Non-HDL-c has also been suggested as a better marker of CVD risk and coronary atherosclerosis^19^ including MetS patients^4^. Moreover, Kilgore *et al*. (2014) found that these patients are more likely to have high non-HDL-c with normal LDL-c rather than high LDL-c with normal non-HDL-c^4^. Our results reveal that in MetS patients when only LDL-c is considered, those at a high CV risk may not be identified as candidates for lifestyle changes or pharmacologic lipid-lowering interventions.

### ApoA1 and ApoB/ApoA1 ratio

ApoA1 has been suggested to mediate the association between MetS and atherosclerosis^20^. Nonetheless, according to the ESC/EAS guidelines, MetS patients show normal ApoA1 concentrations. In our study, we did not find any differences in ApoA1 levels between patients with LDL-c within and above target. Moreover, our results are not in agreement with others which have found reduced ApoA1 levels in MetS patients^21^. Nevertheless, it seems that not only the concentration but also ApoA1 function may be affected in this condition. In fact, Borja *et al*. (2017) have found that in MetS patients, HDL-c/ApoA1 exchange was reduced regardless of ApoA1 levels^21^. ApoB/ApoA1 ratio is a simple and accurate risk factor for CVD^22,23^. In our study, despite the fact that the MetS patients presented a mean ApoB/ApoA1 ratio within the reference range, patients with LDL-c levels above target showed a significantly higher ratio than those within target. Carnevale *et al*. (2011) found that independently from LDL-c, a low ApoB/apoA1 ratio reflects a less atherogenic lipid profile^23^. Also, Wallenfeldt *et al*. (2004) reported that this ratio was associated with an increase in carotid artery intima-media thickness, independently of other risk factors^24^ in MetS patients. Thus, the clinical risk information provided by ApoB/ApoA1 ratio should be recognized.

### Oxidized LDL-c

In our study, 49% of patients with MetS and LDL-c within target had oxidized LDL-c above the reference range. Numerous studies have demonstrated an atherogenic role of oxidized LDL-c in the progression of atherosclerosis^25^ and CHD^26^. Furthermore, the predictive value of oxidized LDL-c seems to be additive to that of the Framingham global risk assessment score for CV risk prediction^26^. Oxidative stress seems to be involved in the pathophysiology of MetS^27^ and oxidized LDL-c is elevated in MetS patients^28^. In fact, MetS dyslipidemia is characterized by the elevation of the easily oxidized sd-LDL particles^29^. This is in accordance with our results where the MetS patients had generally elevated oxidized LDL-c levels and the patients with LDL-c above target showed even higher levels than those with LDL-c within target. More disturbing is the fact that almost half of the LDL-c within target patients presented oxidized LDL-c levels above the reference range, thus suggesting a high risk of progression of atherosclerotic CVD.

### Lp(a) and small and dense LDL particles

Lp(a) is an independent risk factor for CVD^3^ as well as in MetS individuals^30^. Hippe *et al*. (2018) found that in MetS patients treated to obtain LDL-c below 70 mg/dL, carotid atherosclerosis progressed and elevated Lp(a) levels independently predicted this progression^31^. This finding suggests that Lp(a) can also be a useful marker of atherosclerosis evolution in MetS patients. The association of highly atherogenic Sd-LDL particles^32^ with insulin resistance and hypertriglyceridemia suggests its high prevalence in states such as MetS^33^. Our results are in agreement with others reporting an increase of sd-LDL particles in MetS patients^34,35^. Although no differences were observed in sd-LDL particles levels between the two groups, patients with LDL-c above target had a mean plasma sd-LDL particles level above 35 mg/dL indicating a high risk of CHD.

### Lipid parameters correlations

As stated before, concomitant dyslipidemia has a particular pattern characterized by a high flux of free fatty acids, hypertriglyceridemia, low HDL-c values, increased sd-LDL particles and high ApoB values^36,37^. To the best of our knowledge, no studies have evaluated LDL-c correlations with other lipid parameters according to LDL-c status in patients with MetS. As expected, we observed a positive correlation between LDL-c and total cholesterol and non-HDL-c. In accordance with other authors, we also found a positive association of LDL-c with oxidized LDL-c^38^ and with sd-LDL particles^39,40^. Triglycerides affect LDL-c density and thus its propensity to be oxidized whereas HDL-c has a protective role against LDL-c oxidation. Therefore, the atherogenic lipid profile that MetS exhibit can further aggravate oxidative stress in these patients. It is important to note that the presence of sd-LDL particles is affected by other metabolic changes observed in Mets, such as high triglycerides, low HDL-c and insulin-resistance. Non-HDL-c and ApoB were the most strongly correlated with LDL-c. Additionally, non-HDL considered a better risk marker for coronary heart disease exhibited a very strong correlation with ApoB and a strong correlation with oxidized LDL-c. As ApoB and oxidized LDL-c may not be routinely available, the assessment of non-HDL-c could be important to characterize the atherogenic profile. In fact, non-HDL-c is easily obtainable and inexpensive^41^, and represents an appropriate index of CV risk that may be superior to LDL-c^41,42^. In addition, non-HDL-c is not affected by the association between very-low-density lipoprotein (VLDL) cholesterol and triglycerides which is altered in MetS and leads to falsely low LDL values. On the other hand, non-HDL cholesterol represents the cholesterol carried by all of the potentially atherogenic ApoB-containing particles, i.e., VLDL, intermediate-density lipoprotein (IDL), and LDL, chylomicron remnants and lipoprotein(a)^41,42^. Triglyceride-rich lipoproteins, such as VLDL, IDL and their remnants are highly atherogenic^43^. They can be taken up by arterial wall macrophages directly without oxidative modification and, due to their larger size, they carry more cholesterol per particle than LDL^43^. Since MetS is characterized by a postprandial accumulation of triglyceride rich lipoproteins^43^, non-HDL-c may be particularly useful for evaluating CVD risk in patients with this condition.

The logistic regression model showed that higher triglycerides and HDL-c levels appear to increase the odds of LDL-c being within target. Leroux *et al*. (2000) have demonstrated that patients with higher levels of triglycerides are characterized by lower LDL-c levels for any given ApoB concentration compared with subjects with lower levels of triglycerides^44^. An overproduction of the triglycerides-enriched large VLDL causes a high generation of sd-LDL particles that in turn may lead to an overestimation of the VLDL cholesterol and an underestimation of the calculated LDL-c concentrations^45^. Thus, patients with hypertriglyceridemia, such as MetS patients, seem to present lower LDL-c concentrations since these values are falsely diminished by hypertriglyceridemia. Nevertheless, it is important to note that these patients still remain in an environment of high atherogenicity since ApoB remains elevated even when there is a decrease of LDL-c levels^44^. Also, we demonstrated that even when LDL-c levels are optimized, MetS patients still show high levels of important markers of atherogenicity such as non-HDL-c, ApoB and oxidized LDL-c. Cholesterol ester transfer protein is responsible for exchanging the cholesterol esters in the HDL for triglycerides from LDL. Cholesterol ester-rich LDL can return to the liver and be taken up through the LDL receptor^46^. This mechanism may justify the reason why higher HDL-c levels are associated with increased odds of having LDL-c within target. ApoB envelops the surface of several atherogenic lipoproteins^47^ and so greater ApoB values are associated with higher LDL-c levels. MetS patients often present LDL-c/ApoB discordance. In fact, our study suggests that even in patients with “optimal” LDL-c levels ApoB can be elevated.

### Limitations

Our study has some limitations. First, this was a cross-sectional study that did not assess long-term outcomes with respect to the occurrence of CVD and the levels of lipid parameters. Thus, prospective follow-up studies are required to evaluate medical interventions and lipid goal attainment in relation to mortality in patients with MetS. Second, few patients had measurements of sd-LDL particles because it was only recently available in our hospital. Finally, multiple testing can be associated with type 1 errors, even though a Bonferroni correction was applied to the comparisons to counteract this.

### Summary

Our study also has strengths. A small number of studies have assessed non-conventional lipid parameters such as ApoA1, sd LDL particles, Lp(a) or oxidized LDL-c in MetS patients. Our study is an important contribution to the characterization of a comprehensive lipid profile in patients with this condition. Moreover, it also suggests that in MetS patients solely assessing LDL-c could result in missed opportunities for atherosclerotic CVD risk reduction.

## Conclusions

LDL-c reduction has long been the major goal of lipid-modulating therapies; however, patients continue to exhibit CVD-related events. This underscores the need for identification of other predictive lipoprotein parameters. The present study contributes to our understanding of lipid metabolism in MetS patients and it may have important clinical implications. By using LDL-c as the only target, we may be missing significant opportunities to decrease the risk of atherosclerotic CVD. Although further research is needed in this new era, our data suggest that combining LDL-c with non-HDL-c, ApoB and oxidized LDL-c may add important information to better identify therapeutic targets and to stratify CV risk in these patients.

## Data Availability

The datasets generated during and/or analysed during the current study are available from the corresponding author on reasonable request.
